# Two Cases of Foreign Bodies in the Air Passages

**Published:** 1847-06

**Authors:** P. A. Allaire

**Affiliations:** Aurora, Illinois


ARTICLE V.

### *Two Cases of Foreign Bodies in the Air Passages.* By P. A. Allaire, M. D., *of Aurora, Illinois.*

In February, 1843, a little boy of mine, aged two years, was, about 2 o'clock, P. M., whilst eating a piece of cake, suddenly siezed with the usual symptoms of suffocation, which follow the introduction of a foreign body into the trachea. Being absent at the time, a neighbor took the child and thrust her fore-finger deeply into the pharynx, and pushed down, as she stated, the bit of food. The child immediately recovered and resumed its wonted playfulness. On my return, an hour after, I examined the air passages, but could not discover anything unusual. In the evening, about 8 o'clock, the child, while sleeping quietly, coughed slightly, and then was again attacked with apparent fatal suffocation. A physician being next door, was at once called in, who gave an emetic and applied hot cloths to the chest. I suggested opening the larynx, but from the history of the case he supposed it unnecessary. The child expired in about five minutes.

*Post Mortem Examination.*—Every part of the air tube looked natural except a small red spot immediately beneath the epiglottis, where was lodged a common white bean. In consequence of the contact of this body, I judged that spasmodic closure of the valve was the cause of death, for the bean did not more than half fill the passage. It doubtless had been raised from the bronchia by the cough which occurred a few minutes before.

Case II—*February 5th*, 1847.—Was called in haste to see a little boy, Æt. 14 months, who was said to be dying. I was in the house in a few moments, and found the little fellow very comfortable. The mother stated that while playing with some corn a short time before "he had got some down the wrong way and that it had nearly strangled him." I listened to the breathing, and could distinctly hear a clapper-like noise opposite to the upper end of the sternum—the respiration was quite easy. I left the child, and directed that if the suffocation again came on, it should be held up by the feet and struck with the hand between the shoulders.

*February* 6th.—10 o'clock, P. M. Dr. Eastman in consultation. The child has had two attacks of suffocation since last night, and the act of respiration is rather quicker than natural, otherwise very comfortable—no sound now indicates the location of the foreign body, but the efforts made for its expulsion have not succeeded. Under these circumstances, it was decided that the tracheotomy was necessary. After some hesitation the friends consented. The child being very short-necked and fat, I had him secured by an assistant sitting in a chair, the nape of the neck and shoulders were laid on my left knee and the head thus thrown back and firmly held by another assistant. Sitting on the left of the patient, I commenced the incision at the sternum, carried it upwards over the centre of the trachea an inch and a half and carefully cut down to the tube, the child meanwhile screaming and struggling. I then fixed the trachea with a hook, opened it at the centre of the incision, and then introduced a probe-pointed bistoury by which the opening was extended upwards and downwards. After allowing the child a few minutes rest, search was made for the kernel of corn and continued, with short intervals, for an hour without success. The operation, though borne well, left the child somewhat exhausted, and it soon fell asleep. The wound was covered with adhesive straps, which were directed to be removed if suffocation came on. Respiration is carried on mainly through the natural passage, but a little air passes through the strips of dressing.

*February* 7*th.*—6 o'clock, P. M. The last twenty hours have been passed very comfortably. No cough, or suffocation; no symptoms of inflammation; wound looks well. Nothing done except to renew the dressing.

*February* 8*th.*—Afternoon. Dr. Hubbard in consultation. Symptoms of bronchial inflammation have come on during the day, and one fit of suffocation which lasted a few moments this morning. Dr. H. made another search for the foreign body, but was unsuccessful. Prescribed tart. ant. et potass., one eighth of a grain every hour, and poultice to the chest.

*February 9th.*—Bronchitis increased and patient sank at 4 o'clock, P. M.

*Inspection three hours after Death.*—The mucous membrane of the bronchia, from their bifurcation, presented increased redness, with slight effusions of mucus. In the right bronchial tube, about an inch from its origin, was found a kernel of corn, swollen and impacted in the part. The larynx and trachea were normal, except slight redness around the wound.

